# Enzyme-Assisted Discovery of Antioxidant Peptides from Edible Marine Invertebrates: A Review

**DOI:** 10.3390/md15020042

**Published:** 2017-02-16

**Authors:** Tsun-Thai Chai, Yew-Chye Law, Fai-Chu Wong, Se-Kwon Kim

**Affiliations:** 1 Department of Chemical Science, Faculty of Science, Universiti Tunku Abdul Rahman, 31900 Kampar, Malaysia; yew_chye89@hotmail.com (Y.-C.L.); wongfc@utar.edu.my (F.-C.W.); 2 Centre for Bio-diversity Research, Universiti Tunku Abdul Rahman, 31900 Kampar, Malaysia; 3 Department of Marine Bio-Convergence Science, Pukyong National University, 48513 Busan, Korea; sknkim@pknu.ac.kr; 4 Institute for Life Science of Seogo (ILSS), Kolmar Korea Co, 137-876 Seoul, Korea

**Keywords:** antioxidant peptide, enzymatic hydrolysis, marine invertebrate, peptide identification, peptide purification

## Abstract

Marine invertebrates, such as oysters, mussels, clams, scallop, jellyfishes, squids, prawns, sea cucumbers and sea squirts, are consumed as foods. These edible marine invertebrates are sources of potent bioactive peptides. The last two decades have seen a surge of interest in the discovery of antioxidant peptides from edible marine invertebrates. Enzymatic hydrolysis is an efficient strategy commonly used for releasing antioxidant peptides from food proteins. A growing number of antioxidant peptide sequences have been identified from the enzymatic hydrolysates of edible marine invertebrates. Antioxidant peptides have potential applications in food, pharmaceuticals and cosmetics. In this review, we first give a brief overview of the current state of progress of antioxidant peptide research, with special attention to marine antioxidant peptides. We then focus on 22 investigations which identified 32 antioxidant peptides from enzymatic hydrolysates of edible marine invertebrates. Strategies adopted by various research groups in the purification and identification of the antioxidant peptides will be summarized. Structural characteristic of the peptide sequences in relation to their antioxidant activities will be reviewed. Potential applications of the peptide sequences and future research prospects will also be discussed.

## 1. Introduction

Reactive oxygen species (ROS) and reactive nitrogen species (RNS) are free radicals that play vital roles in the body, such as participating in intracellular signaling cascades and host defense against invading pathogens. Imbalance between free radical production and endogenous antioxidant defense may result in cellular oxidative stress, causing oxidative damage to various cellular components, such as DNA, proteins and membrane lipids. Oxidative damage has been implicated in and is believed to be a key factor causing various pathological conditions, such as heart disease, stroke, arteriosclerosis, diabetes, and cancer [1,2,3,4]. Furthermore, accumulation of oxidized proteins underlies the aging process in humans and the development of some age-related diseases [5]. Dietary intake of antioxidants has been associated with reduced risks of some of the aforementioned diseases [6,7]. The effectiveness of antioxidant therapies in preventing and/or managing human pathologies was also highlighted [8,9,10].

Oxidation in the form of lipid peroxidation is also a deleterious process occurring in foodstuffs. Lipid peroxidation is a major cause of rancidity and reduced shelf-life in foods [11]. Oxidation compromises the nutritive value of food, in addition to causing the loss of flavors and the formation of toxic by-products. An effective approach to keep oxidation of food constituents in check is by incorporating synthetic food-grade antioxidants (e.g., butylated hydroxytoluene (BHT), butylated hydroxyanisole (BHA), *tert*-butylhydroquinone (TBHQ), and propyl gallate) during food processing [11,12].

Free radicals can be quenched through a number of mechanisms. Antioxidants can directly scavenge free radicals (e.g., via hydrogen atom transfer or electron transfer) or prevent free radical formation by chelating metal ions. Antioxidants can also interrupt the radical chain reactions of lipid peroxidation, thus retarding its progression. There is currently great interest to search for natural antioxidants as alternatives to the synthetic ones for applications in food processing, functional food development, cosmetic formulations, and therapy. One of the factors driving such a trend is the concern about potential side effects of synthetic antioxidants and consumer preference for natural antioxidants, which are perceived as relatively safe, especially those derived from edible sources [11,12,13,14].

The last two decades have seen a marked increase worldwide in studies searching for bioactive peptides from edible animals and plants as well as from food products and processing wastes derived from them. Bioactive peptides have a broad range of activities, such as antioxidant, antimicrobial, antiviral, antitumor, antihypertensive, immunomodulatory, analgesic, anti-diabetic, and neuroprotective activities [15]. Such bioactive peptides are potential candidates for development into future peptide drugs. The global market for peptide therapeutics was valued at USD 17.5 billion in 2015, expected to hit USD 47 billion by 2025 [16]. There are more than 60 FDA-approved peptide drugs already on the market [17], with about 400 more peptide therapeutics in different phases of preclinical and clinical development as of February 2016 [16]. Overall, peptide drugs are recognized as one of the fastest growing segment, with enormous future growth potential, in the pharmaceutical industry [16,17].

Bioactive peptides are encrypted in an inactive state within the structure of the parent proteins. Such peptides become active after release from the parent proteins, which can be achieved by means of in vitro enzymatic hydrolysis, gastrointestinal digestion, and food processing (e.g., fermentation) [18,19,20]. The activation of antioxidant peptides upon their liberation from the parent protein may be due to their less restricted interaction with free radicals, unhindered by their positions within the bulky protein structure or by poor lipid solubility of the parent protein. The aforementioned proposal, however, remains to be experimentally validated. Enzymatic hydrolysis under optimal conditions is the most efficient and reliable strategy for releasing antioxidant and other bioactive peptides from food proteins, including proteins of the marine origin [21,22,23]. It is also the preferred method for bioactive peptide production in the food and pharmaceutical industries [24]. Antioxidant peptides liberated upon enzymatic hydrolysis of parent proteins are small, ranging from 2 to 20 residues [13,18]. Such peptides usually contain varying percentages of hydrophobic amino acids (e.g., Ala, Leu, and Pro) or aromatic amino acids (e.g., Tyr, His, and Phe) in their sequences. The functionality of antioxidant peptides has been attributed to the ability of such amino acid constituents to donate protons to free radicals, chelate metal ions and/or trap lipid peroxyl radicals [25,26].

Antioxidant peptides are an important area of scientific interest. The input query “antioxidant peptide” OR “antioxidative peptide” in the Scopus database [27], as of November 2016, revealed 542 publications between the years 1992 and 2016. An increasing trend in the number of publications can be seen over the last 24 years, leading to about 80 publications annually between 2013 and 2016 (see Figure S1). A more precise input query “antioxidant peptide” OR “antioxidative peptide” AND “marine” in the Scopus database revealed 22 publications between 2009 and 2016. This comprised of 15 journal article, five book chapters and two reviews. As of September 2016, 531 antioxidant peptide sequences ranging between 2 and 20 residues in length have been deposited into the BIOPEP database [28]. All these point to a growing interest in the research area of antioxidant peptides over the last two decades.

Antioxidant peptides have been isolated and identified from numerous edible marine animals, including various fish species [29,30], and edible marine invertebrates, such as mussels [31,32], clams [33,34], and oysters [35,36]. Identification of antioxidant peptides from food products manufactured from edible marine invertebrates, e.g., fermented mussel sauce [37,38] and shrimp paste [39], has also been reported. The effectiveness of the marine invertebrate peptides in scavenging ROS, chelating metals, suppressing lipid peroxidation, and protecting cells against ROS-induced toxicity has been demonstrated by using chemical and cell-based assays [40,41,42,43]. Notably, the in vivo effects of antioxidant peptides identified from the mussel [44] and sea squirt [45] were reported, suggesting that antioxidant peptides identified from edible marine invertebrates can have biological or physiological significance. Collectively, the aforementioned findings suggest that edible marine invertebrates deserve more attention than they have received to date as a promising source of potent antioxidant peptides.

In reviews on marine bioactive peptides, enzymatic hydrolysates and antioxidant peptides of fish and their processing by-products often overshadowed those of edible marine invertebrates [18,29,46,47]. In this review, we focus on antioxidant peptides purified and identified from enzymatic hydrolysates of edible marine invertebrates, including those prepared by using in vitro gastrointestinal digestion. This review presents an overview of enzyme-assisted production, assay-guided purification, and identification of antioxidant peptides, in addition to their structure–activity relationships. Potential applications of the pure antioxidant peptides in food, therapy and cosmetics are discussed. Future research opportunities, in relation to gaps in current knowledge, are highlighted. When preparing for this review, we analyzed the published antioxidant peptide sequences that were identified from edible marine invertebrates for additional functions (e.g., anticancer activity) or properties (e.g., allergenicity) by using a number of in silico tools. The significance of the new information is discussed in this review where relevant. Our emphasis is on studies which have successfully identified potential antioxidant peptides from edible marine invertebrates. Nonetheless, evidence from protein hydrolysate studies may still be referred to where appropriate. We also highlight studies which have taken the additional step of validating the antioxidant activity of the identified peptide sequences by using synthetic peptides. Literature pertaining to the identification of antioxidant peptides naturally occurring in the cells of edible marine invertebrates, or those present in fermented products (e.g., [38,39]) is not within the scope of this review.

## 2. Enzyme-Assisted Production, Purification, and Identification of Antioxidant Peptides

**Figure 1:**
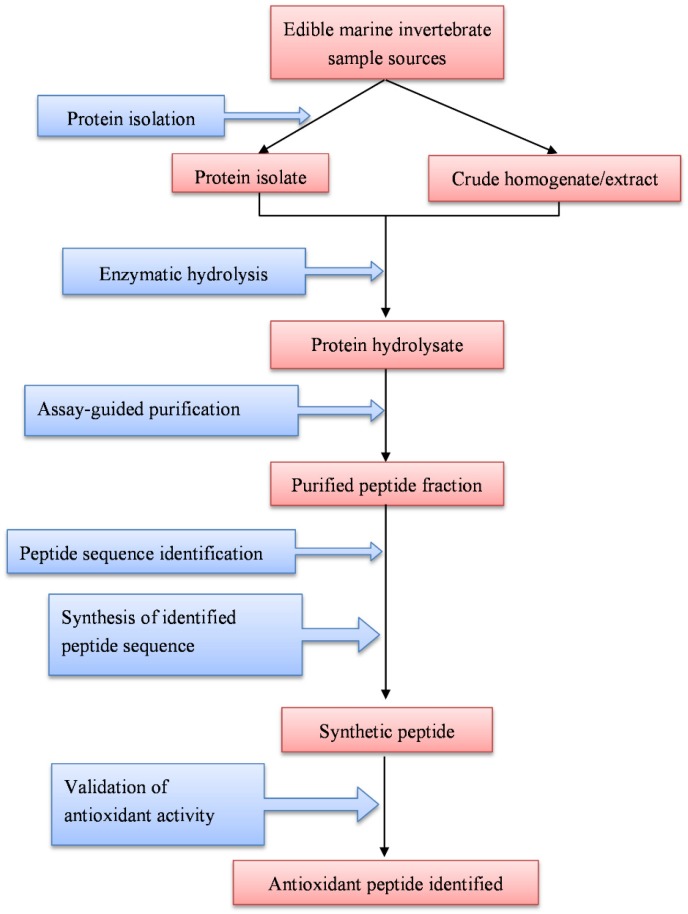
A workflow used for the purification and identification of antioxidant peptides from enzymatic hydrolysates of edible marine invertebrates.

Antioxidant peptides encrypted in the proteins of edible marine invertebrates have been effectively released by enzymatic proteolysis and identified. Table 1 shows the primary structures of 13 edible marine invertebrate-derived antioxidant peptides whose activities were validated using chemically synthesized peptides. A general workflow employed in the purification and identification of antioxidant peptides from edible marine invertebrates is shown in Figure 1.

### 2.1. Production of Antioxidant Peptides

Different forms of protein samples from edible marine invertebrates have been used as the starting material for the isolation of antioxidant peptides. Crude homogenate or mince of samples in cold water as well as pulverized, lyophilized samples, without further protein enrichment, were used for proteolysis and subsequent isolation of antioxidant peptides from the oyster [35], prawn [48], short-neck clam [34], mussels [32,44,52,53], shrimp processing waste [54], scallop female gonad [49], and sea squirt [50]. Isopropanol was used to defat the homogenates of the blood clam [33] and blue mussel [31] prior to their use in the preparation of hydrolysates. To prepare a protein isolate to be used for hydrolysis, Zhou et al. [55] used the trichloroacetic acid/acetone precipitation method to extract proteins from the body wall of the sea cucumber. Nonetheless, most of the aforementioned studies used either crude homogenates or pulverized samples as their starting materials for antioxidant peptide isolation. This suggests that the preparation of a protein isolate or protein-enriched sample is not a requisite for successful isolation of antioxidant peptides.

Enzymatic hydrolysis of protein samples of edible marine invertebrates has been performed by using individual proteases or a combination of digestive proteases with the aim of simulating gastrointestinal digestion. Proteases of animal, plant, and microbial origins that are commercially available in pure form, such as pepsin, trypsin, α-chymotrypsin, papain, alcalase, and neutrase, have been used to prepare protein hydrolysates from edible marine invertebrates. Other commercial proteases that have been used, but less frequently, include kojizyme, flavourzyme, protamex, neutral protease, acid protease, newlase F, pronase and pancreatin [44,45,48,50,53,55].

Owing to the unique cleavage specificities of the proteases, when the same protein sample is treated with different proteases, hydrolysates each consisting of a complex pool of polypeptides hydrolyzed to different extents can be produced. Proteolysis and the generation of various peptides often alter the antioxidant activity of the original protein sample. Hence, one strategy used by some studies was comparing the degree of hydrolysis (DH), yield, and/or antioxidant activity of multiple hydrolysates produced by using different proteases under optimum pH and temperature conditions. A hydrolysate was then chosen and used for the subsequent purification and identification of antioxidant peptides [31,33,41,43,44,45,48,50,53,55,56,57]. For example, Rajapakse, Mendis, Byun and Kim [43] hydrolyzed squid muscle separately with pepsin, trypsin and α-chymotrypsin for six hours. Tryptic hydrolysate, which showed the highest DH and inhibitory activity against linoleic acid oxidation, was selected for further purification. This led to the discovery of two potent antioxidant oligopeptides NADFGLNGLEGLA and NGLEGLK [43]. A number of studies successfully identified antioxidant peptides from optimum hydrolysates chosen based on only antioxidant efficacy, without considering DH or yield (e.g., [31,44,45,53]). Ko, Kim, Jung, Kim, Lee, Son, Kim and Jeon [45] hydrolyzed sea squirt with nine proteases. Based on only relative antioxidant efficacies of the hydrolysates, the authors chose tryptic hydrolysate for further purification; this culminated in the discovery of an antioxidant peptide LPHPSF [45]. Thus, unless it is the objective of the study to evaluate the effectiveness of proteolysis or that availability of protein sources is limited, measurements of DH and yield appear omittable when screening for an optimum hydrolysate for purification of antioxidant peptides.

In contrast to the aforementioned studies, some investigations omitted the tedious process of screening for an optimum hydrolysate, focusing directly on a protein hydrolysate generated by using a single protease. The protease treatments in these studies were chosen based on previously reported efficacy on other species or sample matrices [32,34,35,42,49,51,54]. In these studies, the protease treatments may not be considered optimum for the samples anymore. Even though antioxidant peptides were eventually identified in these studies, the questions remain whether a more potent hydrolysate could have been generated from the samples under investigation, and whether antioxidant peptides of greater potency could have been discovered.

Reports of the use of in vitro gastrointestinal digestion to release antioxidant peptides from proteins of edible marine invertebrates, which culminated in the identification of antioxidant peptide sequences, are scarce. Through the use of pepsin, trypsin and α-chymotrypsin under simulated gastrointestinal conditions and followed by subsequent purification work, antioxidant peptides LVGDEQAVPAVCVP and LKQELEDLLEKQE were identified from the mussel [52] and oyster [40], respectively. The use of a single commercial protease in enzymatic hydrolysis is relatively straightforward when compared with a combination of several proteases, as is the case in in vitro gastrointestinal digestion. Single-protease hydrolysis likely allows a better control of the physicochemical conditions of the process, in addition to that of the compositions and molecular weights of the resulting peptides [58]. For single-protease hydrolysis, proteolysis is carried out only under a selected optimum temperature and pH. Optimum hydrolysis duration is often selected by screening a range of different durations to identify the one which produces the highest DH and/or antioxidant activity (e.g., [31,35,50,56,57]). Often, the *N*- or *C*-terminal amino acid residues of the resulting peptides can be predicted based on the cleavage specificity of the protease used. On the other hand, in vitro gastrointestinal digestion by using proteases requires more complicated pH control and is more time-consuming. For example, simulated gastrointestinal digestion of mussel muscle was performed by first hydrolyzing the sample with pepsin at 37 °C and pH 2.5 for 120 min. Next, the resulting digest was hydrolyzed by trypsin and α-chymotrypsin at 37 °C and pH 7.0 for 150 min [52]. Due to the use of multiple proteases and the unique cleavage specificity of each protease, prediction of the molecular compositions of the resulting peptides is no longer straightforward. Nevertheless, it is believed that purification of antioxidant peptides from simulated gastrointestinal digests may increase the chance of producing peptides that resist breakdown by gastrointestinal peptidases in vivo [40,59]. Notwithstanding this potential advantage, the latter approach is less commonly adopted than the single-protease hydrolysis approach, possibly owing to the aforementioned difficulties.

### 2.2. Purification of Antioxidant Peptides

A protein hydrolysate is essentially a mixture comprising of both antioxidant and prooxidant peptides [60]. Further fractionation will aid in eliminating the prooxidant components [22]. Active hydrolysates produced from edible marine invertebrates were usually subjected to assay-guided fractionation to enrich and eventually isolate the most potent antioxidant peptides. Peptide variability in terms of the molecular mass, net charge and polarity/hydrophobicity underlies the basis of separation techniques used, which include membrane ultrafiltration (UF), low-pressure column chromatography, fast protein liquid chromatography (FPLC), and high-performance liquid chromatography (HPLC). Table 2 summarizes the purification techniques, which enabled successful purification and identification of antioxidant peptides from edible marine invertebrates in various studies.

Membrane UF of protein hydrolysates is often the first step in the assay-guided purification of antioxidant peptides from edible marine invertebrates. This technique uses special porous membranes having certain molecular weight cut-off (MWCO) specifications and made of materials such as regenerated cellulose and polyethersulfone to fractionate peptides in a protein hydrolysate. UF membranes with different MWCO values were frequently used in combination for the fractionation of antioxidant peptides. For example, UF membranes of MWCO 3, 5 and 10 kDa were used to separate the hydrolysates of squid muscle, squid skin gelatin, and the short-necked clam into multiple fractions of different molecular mass ranges [34,41,43]. UF membranes of MWCO 1 and 3 kDa were used to separate a jellyfish protein hydrolysate into three fractions [42]. Less frequently, some studies used only one UF membrane of a selected MWCO for initial fractionation of hydrolysates [33,35,51]. When the antioxidant activities of UF fractions were compared, the fraction with the smallest molecular size, e.g., <1 kDa [33,42] or <3 kDa [31,34,41,43], often showed the strongest activity and was therefore chosen for further purification.

Following membrane UF, the most active fraction obtained from marine invertebrate protein hydrolysates was usually further purified by means of size-exclusion chromatography (SEC) and/or ion exchange chromatography (IEC), either driven by FPLC (e.g., [45,52,56,57]), or carried out as low pressure column chromatography (e.g., [33,34,50]). Notably, some studies omitted the UF step and directly purified protein hydrolysates on an SEC and/or IEC column [45,46,56,57]. Sephadex G-15, G-25 and G-50 are SEC stationary phases that have been used in the purification of antioxidant peptides from marine invertebrate hydrolysates. Sephadex G-15 was used in the purification of antioxidant peptides from the oyster [35] and blue mussel [31]. Sephadex G-25 was used more commonly by other researchers [33,34,41,43,45,48,49,50,54,56,57]. Zhou, Wang and Jiang [55] used both Sephadex G-25 and G-50 stationary phases to purify antioxidant peptides from the sea cucumber. Elution of SEC columns was usually performed by using deionized or distilled water and monitored at 214 nm [36], 220 nm [33,34,49,55], or 280 nm [31,45,54,56,57].

IEC can be divided into two broad classes based on the type of exchangers used: anion exchange chromatography (AEC) and cation exchange chromatography (CEC). For the purification of antioxidant peptides by AEC from the Indian squid [57], shortclub cuttlefish [56], oyster [40], mussel [44,52], sea cucumber [55], short-necked clam [53], blood clam [33], sea squirt [45], and jellyfish [42], weak anion exchangers with diethylaminoethyl (DEAE) exchange groups were used. To purify antioxidant peptides by CEC from the prawn [48], squid [43], shrimp processing by-products [54], and jumbo squid skin gelatin [41], resins with sulphopropyl (SP) strong cation exchange groups were used. Prior to IEC, Zhao, Huang and Jiang [54] extracted lyophilized hydrolysate of shrimp processing by-products with 90% methanol overnight to remove interfering non-protein compounds having high antioxidant activity. Most of the aforementioned studies eluted peptide fractions during AEC and CEC by using a linear NaCl gradient, although step gradient elution with NaCl was also reported for the purification of antioxidant peptides from the blood clam and sea cucumber [33,55]. Elution profiles of IEC were monitored in these studies at 215 nm [43], 220 nm [33,41,55], and 280 nm [40,44,45,53,54,56,57].

Some researchers purified their peptide samples on only SEC [34,50], whereas others on only IEC [32,40], before proceeding to peptide purification by Reversed-Phase HPLC (RP-HPLC). The use of both SEC and IEC to purify antioxidant peptides from the protein hydrolysates of the prawn [48], Indian squid [57], shortclub cuttlefish [56], sea squirt [45], and shrimp processing by-products [54] has also been reported. When purifying antioxidant peptides from the sea cucumber, Zhou, Wang and Jiang [55] adopted the approach of two-step SEC linked by IEC. Their peptide sample was purified on a Sephadex G-50 column, followed by a DEAE cellulose DE-52 column, and lastly on a Sephadex G-25 column, guided by OH^•^ and O_2_^•−^ scavenging assays. The Sephadex G-25 step allowed them to desalt their sample prior to subjecting it to RP-HPLC separation [55].

RP-HPLC was almost always the last purification technique performed before the peptides purified from edible marine invertebrates were taken to peptide sequence determination. Peptide samples were often purified with low-pressure column chromatography before they were subjected to RP-HPLC purification. Notwithstanding, Asha, Remya Kumari, Ashok Kumar, Chatterjee, Anandan and Mathew [36] purified an active UF fraction of the oyster protein hydrolysate with four C18 solid phase extraction (SPE) cartridges connected in series prior to subjecting it to RP-HPLC purification. With a few exceptions [49,56,57], most studies which successfully identified antioxidant peptides from edible marine invertebrates purified their peptides by RP-HPLC at least once. RP-HPLC was carried out using a C18 stationary phase with a linear gradient of acetonitrile either containing low percentage of formic acid [51], or trifluoroacetic acid (TFA)(e.g., [31,32,33,36,45,54]), or without any mobile-phase modifiers [34,41,43,44,50,52,53]. An alternative mobile phase comprising of a linear gradient of methanol with 0.1% TFA was reported by others who successfully purified and identified antioxidant peptides from the jellyfish [42] and sea cucumber [55]. Eluted RP-HPLC fractions were monitored at 214 nm [36,54,55], 215 nm [32,40,41,43,44,52,53], 220 nm [33,34,42,48,50], 230 nm [35], or 280 nm [31,35,45,61].

Multi-step RP-HPLC involving the use of a semi-preparative C18 column followed by one or two analytical C18 columns was used to purify antioxidant peptides from the short-necked clam [53], giant squid [43], oyster [40], hard-shelled mussel [44], and shrimp processing by-products [54]. To isolate antioxidant peptides from the blue mussel, Park, Kim, Ahn and Je [32] purified their sample on the same semi-preparative C18 column twice, each time using a different linear acetonitrile gradient.

### 2.3. Identification of Antioxidant Peptides

Following RP-HPLC purification, the purified antioxidant peptides were usually taken to amino acid sequence identification by using either liquid chromatography-tandem mass spectrometry (LC-MS/MS) or the Edman degradation method. Typical LC-MS/MS experiments which enabled successful identification of antioxidant peptides from edible marine invertebrates involved the use of a quadrupole time-of-flight tandem mass spectrometer, equipped with an electrospray ionization (ESI) source and run in the positive ion mode (e.g., [42,49]). Alternatively, the use of a hybrid triple quadrupole/linear ion trap mass spectrometer to sequence antioxidant peptides from the oyster [36], shortclub cuttlefish [56], and Indian squid [57] was reported. To identify a radical scavenging peptide from shrimp processing by-products, Zhao, Huang and Jiang [54] used an ESI-triple quadrupole mass spectrometer run in the negative ion mode. Mass spectra data obtained were typically analyzed with de novo sequencing algorithms to identify the amino acid sequences of the peptides isolated. At the same time, information on molecular mass of the isolated peptide can be computed from the mass spectra data [32,42,55].

On the other hand, using a protein sequencer, the sequencing of antioxidant peptides purified from the short-necked clam [34,53], hard-shelled mussel [44], blue mussel [31], blood clam [33], and prawn [48] was carried out based on the Edman degradation reaction. Following peptide sequencing, mass spectrometry was used by some research groups to determine the molecular masses of the antioxidant peptides identified [31,33,48]. The use of SEC to further purify the antioxidant peptide contained in an active RP-HPLC fraction prior to determining the amino acid sequence of the peptide by using the Edman degradation method was also reported [44,53].

After an antioxidant peptide is identified, synthesizing the peptide and validating its bioactivity will provide valuable confirmation of the function of the antioxidant peptide. A search of the literature revealed that of the 22 reports published in the last 16 years of antioxidant peptides identified from edible marine invertebrates, only seven studies have validated the antioxidant peptides identified with chemically synthesized peptides [32,42,45,48,49,50,51]. For synthetic peptide production, Fmoc-based solid-phase peptide synthesis was typically carried out. Purity of the synthetic peptide and its molecular mass were analyzed by means of RP-HPLC and mass spectrometry [32,45,49,51].

## 3. Evaluation of Antioxidant Activities

Different types of chemical and cell-based assays have been used in the assay-guided purification and the characterization of antioxidant peptides from edible marine invertebrates (Table 2). Among the chemical assays used, radical scavenging and lipid peroxidation inhibition assays were common. Concerning the principles as well as the advantages and limitations of the antioxidant assays listed in Table 2, we refer the reader to reviews by Zhong and Shahidi [11], Sila and Bougatef [18], Samaranayaka and Li-Chan [21], and Wu, et al. [46].

The 2,2-diphenyl-1-picrylhydrazyl (DPPH) radical scavenging assay, likely due to its simplicity, was used in quite a number of studies to guide the purification of antioxidant peptides from the oyster [35,36], mussel [31,32], blood clam [33], short-necked clam [34], scallop female gonad [49], shortclub cuttlefish [56], Indian squid [57], and shrimp processing by-products [54]. Besides DPPH scavenging activity, the ability of antioxidant peptides identified from edible marine invertebrates to quench ABTS, OH^•^, O_2_^•−^, alkyl, peroxyl, and carbon-centered radicals have been reported (Table 2). Using a linoleic acid model system, the ability of antioxidant peptides identified from the oyster [40], mussel [31,52], blood clam [33], squid [41,43], shortclub cuttlefish [56], Indian squid [57], and prawn [48] to inhibit lipid peroxidation has also been demonstrated. The ability to protect against OH^•^-induced DNA damage has been demonstrated for oyster-derived peptide LKQELEDLLEKQE [40], short-necked clam-derived GDQQK [34], scallop-derived HMSY and PEASY [49], Indian squid-derived WCTSVS [57], and shortclub cuttlefish-derived I/L N I/L CCN [56]. Using the fluorescence probe DCFH-DA, cellular radical scavenging activity of LKQELEDLLEKQE and LPHPSF in mouse macrophage cells (RAW 264.7) [40,45] and that of WCTSVS in human breast adenocarcinoma cells (MCF7) [57] has been demonstrated.

Antioxidant peptides identified from the giant squid [43], blue mussel [32], jellyfish [42], sea squirt [45], and jumbo squid skin gelatin [41] were shown to mitigate radical-induced cytotoxicity (Table 2). Cell types used in these studies were human lung fibroblast cells [41,43], Chang liver cells [32], RAW 264.7 [45], and rat cerebral microvascular endothelial cells (RCMEC) [42]. Notably, the cytoprotective activity of PIIVYWK, FSVVPSPK, VKP, and VKCFR was confirmed using chemically synthesized peptide sequences, rather than purified fractions [32,42]. Mendis, Rajapakse, Byun and Kim [41] found that the efficacy of FDSGPAGVL and NGPLQAGQPGER in protecting human lung cells against *t*-butyl hydroperoxide-induced oxidative cell death was comparable to or surpassed that of α-tocopherol. On the other hand, the concentration range of Indian squid-derived WCTSVS that was non-toxic to MCF7 cells [57], of shortclub cuttlefish-derived I/L N I/L CCN non-toxic to human colorectal adenocarcinoma cells (HT29) [56], and of sea squirt-derived LPHPSF non-toxic to RAW 264.7 cells [45] have been reported. LKQELEDLLEKQE purified from the oyster was also reportedly non-toxic to human embryonic lung fibroblast and RAW 264.7 cells, although the range of peptide concentration tested was not reported [40].

**Figure 2:**
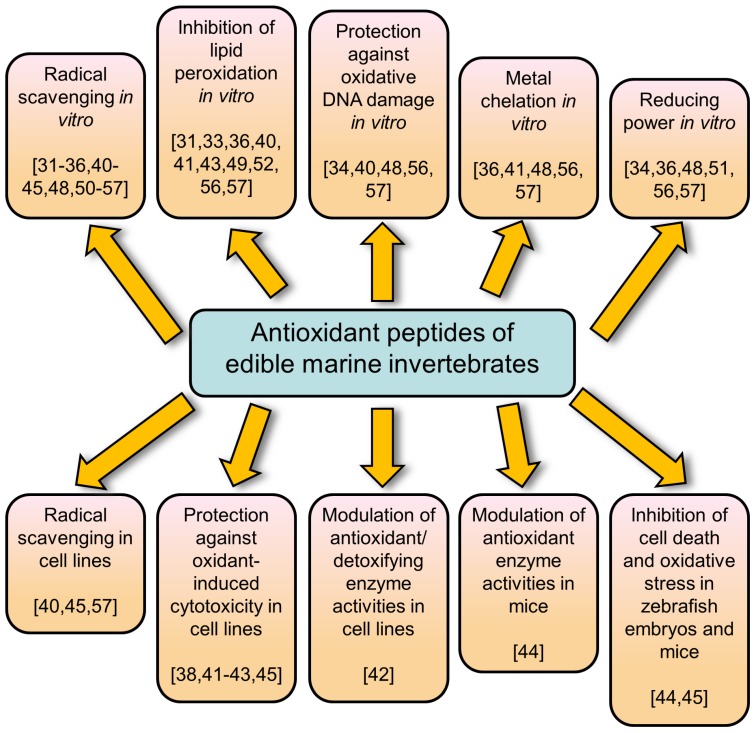
Antioxidant mechanisms reported for antioxidant peptides identified from edible marine invertebrates.

Little work has been done to elucidate the molecular or biochemical basis underlying the cytoprotective effects of antioxidant peptides identified from edible marine invertebrates. The two peptides PIIVYWK and FSVVPSPK derived from the blue mussel protected human liver cells against H_2_O_2_-induced toxicity by upregulating the protein expression of hemeoxygenase-1 [32]. Protection of RCMEC cells by jellyfish-derived peptides VKP and VKCFR against H_2_O_2_-induced toxicity, on the other hand, was associated with enhanced enzymatic activities of superoxide dismutase, catalase, and glutathione peroxidase [42]. Current evidence is preliminary, but the observed cytoprotection appears attributable to the ability of the antioxidant peptides to activate the expression of cytoprotective enzymes. To the authors’ knowledge, only two animal studies have been conducted to investigate the effects of antioxidant peptides identified from any edible marine invertebrates. Oral administration of peptide SLPIGLMIAM to mice was reported to inhibit the level of malondialdehyde in the liver, thus providing evidence for in vivo antioxidant effect [44]. The treatment enhanced superoxide dismutase activity in vivo but had no effects on catalase or glutathione peroxidase activities [44]. Recently, sea squirt-derived antioxidant peptide LPHPSF was shown to be capable of attenuating radical-induced cell death and ROS production in zebrafish embryos [45]. Figure 2 summarizes modes of action reported for antioxidant peptides identified from edible marine invertebrates.

Owing to the diverse types of assays and assay conditions used to characterize the bioactivities of food-derived antioxidant peptides, comparison between studies is often difficult [21]. For example, Zhou, Wang and Jiang [55] and Sudhakar and Nazeer [57] reported markedly different levels of BHT potency in their hydroxyl radical and superoxide anion radical scavenging assays. Such a discrepancy is likely due to the different assay protocols used in the two studies. Chi et al. [33] compared the EC_50_ values of blood clam-derived WPP for DPPH and ABTS scavenging activities with EC_50_ values reported for other antioxidant peptides by other research groups. Such a comparison will be more meaningful if identical assay conditions and ideally an identical positive control were established between studies to check for laboratory-to-laboratory variations. Although Chi et al. [33] and Zhuang et al. [62] both ran the DPPH and ABTS scavenging assays using ascorbic acid as the positive control, comparison of relative potency of peptides between studies remains difficult because EC_50_ for ascorbic acid was reported by only Zhuang et al. [62]. Comparison of antioxidant potential between peptides based on data obtained from different antioxidant assay procedures in different studies (e.g., [31,32]) should therefore be considered with caution.

## 4. Molecular Characteristics and Structure–Activity Relationship

The structure–activity relationships (SAR) of food-derived antioxidant peptides were recently reviewed [18,25,26]. Comprehension of SAR may contribute towards an effective prediction of potential antioxidant activity in a new peptide and the development of strategies for enzyme-assisted release of antioxidant peptides from food proteins [18]. Generally, structural characteristics such as molecular mass, hydrophobicity, amino acid composition, and peptide sequence are considered determinants of the antioxidant activity of a peptide [25,26]. Despite such generalizations, current knowledge of the SAR of antioxidant peptides is still incomplete.

In the context of molecular mass, a majority of the food-derived antioxidant peptides have molecular masses ranging between 500 and 1800 Da [21]. Concurring with this, we found that 26 of the 32 antioxidant peptides identified from edible marine invertebrates fall within this range (Table 3). Peptides having smaller molecular masses are generally believed to have greater antioxidant activity than those having larger masses [25,26]. In accordance with this, during the fractionation of marine invertebrate protein hydrolysates with UF membranes, peptidic fractions having the lowest molecular mass range showed the highest DPPH radical scavenging activity [31,33,36] and the highest hydroxyl radical scavenging activity [42,53]. Similarly, UF fractions with the lowest molecular mass range (<3 kDa), which were prepared from the giant squid muscle [43] and jumbo squid skin gelatin [41], had the highest inhibitory activity against linoleic acid oxidation relative to other UF fractions in the same study. By contrast, in SEC or gel filtration chromatography, the peptidic fraction with the lowest molecular mass was not always the most potent antioxidant fraction. SEC fractions with the lowest molecular mass were reported to be the most active fraction in scavenging DPPH [54,57], hydroxyl [35,57], and peroxyl [50] radicals, as well as having the highest reducing power [57]. Furthermore, others reported that SEC fractions with intermediate molecular masses were the most active in scavenging DPPH [31,33,34,49,56] and ABTS [51], as well as having the strongest reducing power [34,51,56]. Zhou, Wang and Jiang [55] had purified antioxidant peptides from a sea cucumber protein hydrolysate by using a two-step SEC. In the first step, the fraction with the lowest molecular mass showed the highest superoxide and hydroxyl scavenging activity. In the second step, the fraction with the highest molecular mass was the most potent [55]. Alemán et al. [51] suggested that in SEC, a peptidic fraction with the lowest molecular mass may contain large number of free amino acids and small peptides lacking antioxidant activity. Taken together, the aforementioned discrepancies imply that the notion of smaller peptides exhibiting greater antioxidant activity may be oversimplified, or is only applicable to the analysis of protein hydrolysates purified by certain techniques. The proposal thus contributes only limited, if any, knowledge about the SAR of antioxidant peptides.

Hydrophobicity is an important determinant of antioxidant activity of peptides. The presence of hydrophobic residues allows an antioxidant peptide to interact with lipid-soluble free radicals and retard lipid peroxidation [64]. In agreement with this, 15 antioxidant peptides identified from edible marine invertebrates, which inhibited lipid peroxidation in vitro, contain 28%–100% hydrophobic residues in their sequences (Table 4). In fact, the 12 antioxidant peptides identified from edible marine invertebrates, whose antioxidant activities (radical scavenging, reducing power, and inhibition of lipid peroxidation) were validated by using synthetic peptides, contain 22%–71% hydrophobic residues (Table 5). Alemán et al. [51] compared the reducing power and ABTS radical scavenging activity of three peptides having the same molecular mass, namely GPLGLLGFLGPLGLS, GPOGOOGFOGPOGOS (where O represents hydroxyproline), and GPOGOOGFLGPOGOS. The peptide GPLGLLGFLGPLGLS, the most hydrophobic sequence of the three, was found to have the strongest antioxidant activity [51]. Apparently, hydrophobicity may confer antioxidant activity to peptides not only in a lipid oxidation model, but also via other antioxidant mechanisms.

Amino acid compositions and peptide sequences also influence the antioxidant activity of a peptide [25,26]. Food-derived antioxidant peptides often contain hydrophobic residues at the N-terminus, in addition to having Pro, His, Tyr, Trp, Met, and Cys in the peptide sequences [21]. Examination of the list of 32 antioxidant peptides identified from edible marine invertebrates (Table 3) revealed 18 sequences containing a hydrophobic residue at the N-terminus (FDSGPAGVL, FIKK, FKK, FSVVPSPK, I/L N I/L CCN, IKK, ISIGGQPAGRIVM, LKQELEDLLEKQE, LPHPSF, LVGDEQAVPAVCVP, LWHTH, PEASY, PIIVYWK, PVMGA, VKCFR, VKP, WCTSVS, and WPP). The 14 other peptides in Table 3, although lacking a hydrophobic N-terminal residue, contain at least one hydrophobic residue in their sequences. The ability of some of the aforementioned peptides to inhibit lipid peroxidation (Table 4) may therefore be attributable, at least in part, to the presence of hydrophobic residues. GDQQK identified from the short-necked clam is the only antioxidant peptide which contains no hydrophobic residue in its sequence. At present there is no report of GDQQK having any lipid peroxidation inhibitory activity [34].

His-containing peptides can exert their antioxidant effects through the hydrogen donating, lipid peroxyl radical trapping, and metal ion chelating actions of the His imidazole group [66]. On this score, the presence of His residue may account for, to a certain extent, the radical scavenging activities exhibited by QHGV [35], LWHTH [50], SVAMLFH [54], LPHPSF [45] and HMSY [49]. On the other hand, aromatic amino acid residues (e.g., Tyr, Trp and Phe) in peptides may exert antioxidant effects by donating protons to electron-deficient free radicals [26,67]. Thus, the presence of aromatic amino acid residues may account for the antioxidant activities demonstrated by some peptides identified from edible marine invertebrates (e.g., WPP, FKK, and FIKK).

IKK (388 Da) and FKK (422 Da), two antioxidant tripeptides identified from the prawn, showed different levels of inhibition against linoleic acid oxidation despite both containing one hydrophobic residue. Furthermore, a mixture of the constituent amino acids at the same concentrations as the peptides showed no antioxidant activity [48]. This suggests that the specific amino acid sequences, and more specifically, the identity of the N-terminal residue of the tripeptides, are key to their potency as antioxidants. Wu et al. [49] compared the antioxidant activity of two peptides of similar molecular masses that were identified from scallop female gonads, namely HMSY (536 Da) and PEASY (565 Da). HMSY, despite its lower content of hydrophobic residues (25%), exhibited 4.6-fold stronger hydroxyl radical scavenging activity than PEASY (40% hydrophobic residues) [49]. It was suggested that antioxidant activity of the two peptides may be attributed to the Tyr residue at the C-terminus of the peptides [49]. Nevertheless, considering their distinct difference in antioxidant potency, the specific amino acid sequences in HMSY and PEASY may have imparted different levels of antioxidant effects too.

Several antioxidant peptides identified from edible marine invertebrates which exhibited lipid peroxidation inhibition activity (Table 4) were amphiphilic in nature. For example, NGLEGLK, NGPLQAGQPGER, and FIKK are antioxidant peptides containing one or more hydrophilic, basic amino acids (e.g., K and R), in addition to varying percentages of hydrophobic residues in their sequences. The lipophilic and hydrophilic amino acid residues may have collectively contributed to the overall antioxidant activity of the peptides. It was noted that the amphiphilic nature of peptides may influence their antioxidant activity by facilitating their interaction with a hydrophobic target and also proton exchanges with free radicals [26]. Amphiphilic peptides may reside in the oil-water interface and effectively quench free radicals in both the aqueous and oil phases of a linoleic acid emulsion system [68].

Altogether, despite others’ assertions [25,26], molecular mass is not a reliable determinant of antioxidant activity of peptide fractions derived from edible marine invertebrates. On the other hand, our assessment of the hydrophobicity and amino acid compositions of the antioxidant peptides identified from edible marine invertebrates supports the proposal that these two parameters are important determinants of peptide antioxidant efficacy [25,26]. Thus, hydrophobicity and amino acid compositions are two criteria that are useful to future efforts to develop strategies to discover antioxidant peptides from edible marine invertebrates based on SAR knowledge.

## 5. Potential Applications in Food, Therapy and Cosmetics

The bioactive peptide ingredient market, currently dominated by the soy and dairy industries, is very competitive [69]. Bioactive peptides, in pure form and as an unpurified mixture, have been incorporated into a number of functional foods and dietary supplements already on the market [64,69,70,71]. At present, a number of peptides with well-established antioxidant effects are commercially available as dietary supplements, e.g., reduced glutathione, carnosine, anserine, and melatonin. Thus there is market demand for antioxidant peptide-based supplements or functional foods. Nevertheless, marine peptide-based functional foods and dietary supplements with approved antioxidant health claims are scarce [12,72]. To the authors’ knowledge, health foods or supplements containing pure antioxidant peptides identified from edible marine invertebrates are either unavailable or have not been documented in the literature. Apparently, opportunities abound for the development of novel foods or dietary supplements containing antioxidant peptides identified from edible marine invertebrates. On this score, peptides which exhibited antioxidant potential in cell culture models and in mice, for example, VKP and VKCFR identified from the jellyfish [42] and SLPIGLMIAM identified from the mussel [44] are promising candidates for the development of high-value peptide ingredients for health food or supplements. Furthermore, VKP and VKCFR which demonstrated antioxidant and angiotensin converting enzyme inhibitory activities [42] as well as WPP which showed antioxidant and antiproliferative potential [33] are valuable candidates for the development of multifunctional health foods or supplements.

Antioxidant peptides which can inhibit lipid oxidation are potentially useful in the preservation of lipid-rich foods [18]. Besides food quality preservation during storage, antioxidant peptides incorporated into foodstuffs can provide nutrients in the form of amino acids when consumed, which is an advantage over synthetic antioxidants. Among the antioxidant peptides identified from edible marine invertebrates, 15 of them inhibited in vitro lipid oxidation (Table 4). Peptides isolated from the giant squid (NADFGLNGLEGLA, and NGLEGLK) [43], mussel (LVGDEQAVPAVCVP) [52], and oyster (LKQELEDLLEKQE) [40], for instance, were comparable or superior to lipid-soluble antioxidant alpha-tocopherol in inhibiting lipid peroxidation. Tripeptide WPP isolated from blood clam protein hydrolysate was as effective as glutathione in attenuating lipid peroxidation [33]. Among the 15 peptides, prawn muscle-derived IKK, FKK, and FIKK are the most notable. Their ability to dampen lipid peroxidation, validated using chemically synthesized peptides designed based on the identified sequences, surpassed that of alpha-tocopherol [48]. Currently, the antioxidant effects of edible marine invertebrate-derived peptides in food systems are a gap in knowledge. Nevertheless, some work has been done using protein hydrolysates prepared from edible marine invertebrates. Squid protein hydrolysate prepared using papain was shown to retard lipid oxidation in a sardine mince model system as effectively as ascorbic acid [73]. Cuttlefish skin gelatin hydrolysate prepared with alcalase delayed lipid oxidation in cooked turkey meat sausage during storage at 4 °C [74]. Protein hydrolysates prepared by alcalase hydrolysis of shrimp waste, when applied as a dipping treatment on whole Croaker fish, reduced lipid oxidation and maintained fish skin color during storage at 4 °C for 10 days [75].

Recently, the peptide PAGT isolated from Amur sturgeon skin gelatin was reported to inhibit lipid oxidation when added to the Japanese sea bass mince [76] and to retard both lipid and protein oxidation when applied in combination with caffeic acid to the same mince model [77]. These studies [76,77] suggest that besides protein hydrolysates, pure antioxidant peptides may be developed into novel food additives to be used for delaying oxidative rancidity and maintain food quality. Edible marine invertebrate-derived peptides with the ability to inhibit lipid oxidation in vitro (Table 2) have potential applications as food preservatives, but future work is required to first investigate their efficacy in food systems. Furthermore, issues such as potential unfavorable effects on food organoleptic properties and antioxidant peptide stability following food processing operations (e.g., heat treatment) have to be addressed [21]. To make their application as food antioxidant additives economically feasible, it is crucial that such peptides have considerably higher efficacy than crude protein hydrolysates, thus allowing the peptides to be used at low quantities (0.001%–0.02%) [18]. Otherwise, the use of crude protein hydrolysates, e.g., those prepared from the squid, cuttlefish skin gelatin and shrimp waste [73,74,75], are likely more cost-effective options as food antioxidant additives. Moreover, certain aromatic and hydrophobic amino acids that confer antioxidant activity on peptides may impart bitterness. Antioxidant peptides having high potency can be added to foodstuffs in small quantities, thus minimizing the issue of food bitterness [78].

Antioxidant peptides have promising future applications as therapeutics or adjunct therapeutics against oxidative damage-related diseases or conditions. In rats, the ability of a mitochondrion-targeted antioxidant peptide SS31 to reduce myocardial lipid peroxidation and infarct size in ischemia-reperfusion injury was reported [79]. The peptide also mitigated kidney injury in diabetic mice [80] and retinal damage in diabetic rats [81]. In a mouse model of burn injury, the antioxidant peptide SS31 alleviated symptoms of burn injury and promoted recovery of skeletal muscle mitochondrial functions [82,83]. An antioxidant peptide identified from ostrich egg white protein hydrolysate was also shown to promote wound healing in rats [84]. At present, the effects of pure antioxidant peptides identified from edible marine invertebrates have not been tested in animal models of human diseases. Future research in this direction should provide a better understanding of the potential of edible marine invertebrate-derived antioxidant peptides as therapeutic agents for targeting oxidative stress-related diseases or conditions. One current focus of the pharmaceutical industry is the development of peptide drugs having multiple pharmacological activities [17]. On this score, some antioxidant peptides identified from edible marine invertebrates (Table 2) may have potential applications in the development of future multifunctional peptide therapeutics and/or adjunct drugs. Promising candidates include blood clam-derived WPP, which in addition to its antioxidant activity, also inhibited the proliferation of different cancer cell lines, showing only marginal cytotoxicity against normal cells [33]. Other multifunctional antioxidant peptides discovered from edible marine invertebrates are VKP and VKCFR (jellyfish) [42] and GPLGLLGFLGPLGLS (squid skin gelatin) [51] which exhibited angiotensin converting enzyme inhibitory activity.

Skin care or cosmetic products are one area in which antioxidant peptides discovered from edible marine invertebrates may have applications. The beneficial effects of bioactive peptides whether as cosmeceuticals or as dermatologic tools which modulate collagen, elastin and melanin synthesis have been highlighted [85,86,87,88]. The observations that more than 25 different peptides are used as active ingredients in skin care products manufactured by companies based in Canada, USA, Spain, Switzerland, and France, and that many more skin care-relevant peptides are in development worldwide point to the commercial value of peptides as cosmeceutical ingredients [88]. Antioxidant peptides, exemplified by glutathione and carnosine, are one of the many bioactive peptides used in skin care products. One key consideration in using peptides in the formulation of skin care products is skin penetration. Longer peptides (e.g., containing six or more amino acids) generally do not penetrate well into deeper layers of the skin [85]. Longer peptides are also likely more expensive to produce compared to the shorter ones. Hence, among the antioxidant peptides derived from edible marine invertebrates (Table 2), those with short sequences (e.g., QHGV [35], WPP, QP [33], HMSY [49], VKP [42], IKK, FKK, and FIKK [48]) may have greater potential for application in skin care products compared with the rest. Such short peptides can be used as lead structures for the development of effective peptide ingredients taking into considerations factors such as potency, transcutaneous delivery, stability and compatibility within cosmetic formulations, toxicity, and cost of production [85,88]. In the light of many bioactive peptides used currently in skin care formulations, antioxidant peptides identified from edible marine invertebrates may have to outperform current peptide ingredients in critical areas, such as cost of production, potency and multifunctionality, in order to stand out as candidates for future peptide ingredients. The copper-binding tripeptide GHK-Cu, which is popularly used in skin care products, is an antioxidant peptide with wound healing, anti-inflammatory, and anti-aging properties [89]. Glutathione, in addition to its well-established antioxidant properties, also exhibits skin-lightening effects [90]. Thus, the multifunctional nature of the antioxidant peptides identified from edible marine invertebrates (Table 2) warrants more research.

## 6. Current Gaps in Knowledge and Future Perspectives

### 6.1. Biological Significance

One issue to take note of in future research is the biological significance of edible marine invertebrate-derived antioxidant peptides. Among the 32 antioxidant peptides identified from edible marine invertebrates (Table 2), three peptides showed cellular radical scavenging activity [40,45,57]; nine peptides, protective effects against AAPH-, H_2_O_2_- or *t*-butyl hydroperoxide-induced cytotoxicity [32,41,42,43,45]; and two peptides, in vivo antioxidant effects [44,45]. It is unclear whether antioxidant effects of the other 19 peptides, discovered by using cell-free or chemical-based methods, have any biological significance. Shen et al. [91] identified 16 antioxidant peptides from ovotransferrin guided by the oxygen radical absorbance capacity (ORAC) assay; antioxidant activity of pure peptides synthesized based on the 16 sequences was validated with the ORAC assay. Recently, Jahandideh et al. [92] reported that these 16 peptides had no antioxidant activities in human umbilical vein endothelial cells. A plausible explanation would be that a chemical- or cell-free antioxidant assay, such as the ORAC assay, fails to reflect the complexity of a biological system when used to assess antioxidant peptides [92]. Whether the same observation will hold had a different cellular antioxidant assay or cell type been used to test the 16 peptides in the study of Jahandideh, Chakrabarti, Davidge and Wu [92] is an open question. In any case, if the targeted applications of an edible marine invertebrate-derived antioxidant peptide involve a biological system, it is highly desirable to have its biological significance ascertained at least in a cell culture model before more time and effort are invested to studying it.

### 6.2. Stability

Depending on the intended applications for an antioxidant peptide, its stability upon thermal processing and gastrointestinal digestion, its bioactivity in biological tissues or cells, as well as its bioavailability may or may not be crucial. For example, antioxidant peptides to be used for food preservation may not need to exhibit significant cellular radical scavenging activity or gastrointestinal stability, but should remain active when incorporated into food matrices and ideally be thermal-stable. Meanwhile, antioxidant peptides to be used as injectable therapeutics may not need to be thermal-stable or resistant to gastrointestinal digestion, but should not be readily degraded by human plasma peptidases.

To gauge the potential of an antioxidant peptide as orally administered therapeutic agent or as functional food ingredient, the intestinal stability and absorption of the peptide is a key consideration. Monolayers of the human colorectal adenocarcinoma cell (Caco-2) are considered a model of the intestinal epithelium. The Caco-2 permeability assay has been used to demonstrate that CKYVCTCKMS, an antioxidant peptide derived from buffalo milk product, had good stability against brush-border peptidases and was absorbed intact through a Caco-2 monolayer [93]. On the other hand, the in vitro resistance of a lactoferricin B-derived antihypertensive peptide RRWQWR to human plasma peptidases has also been investigated through monitoring their degradation in human serum over time with RP-HPLC [94]. At present, very little is known about the in vivo stability of the antioxidant peptides identified from edible marine invertebrates. Future studies to investigate their bioavailability and stability against intestinal and plasma peptidases are warranted to better appraise their potential applications in biological systems. Certain strategies may be adopted to enhance the intestinal and systemic stability of the edible marine invertebrate-derived antioxidant peptides if necessary. Peptide structural modifications such as cyclisation and replacement of l-amino acid with d-amino acid as well as other related strategies have been reviewed [95,96]. Proline- and hydroxyproline-containing peptides are generally able to resist breakdown by digestive enzymes [97]. On this score, selection of proline-containing peptides from the pool of antioxidant peptides identified from edible marine invertebrates for further study or incorporation of a proline residue into a selected antioxidant peptide may be a promising approach.

Development of efficacious and stable peptide drugs that can be administered orally, thus improving patient convenience, is one of the current foci of the pharmaceutical industry [17]. Notwithstanding, a pertinent point to consider is that in vivo absorption of an antioxidant peptide may not always be required for its application. Ingested materials and pathogens can induce gastrointestinal oxidative injury and inflammation, which may increase the risks of diseases such as peptic ulcers, cancers, and inflammatory bowel disease [98]. Hence, antioxidant peptides that are resistant to gastrointestinal proteolysis following oral administration, even without getting absorbed, may still play a role in maintaining the health of the epithelial lining of the gastrointestinal tract [22].

### 6.3. Application of In Silico Tools

Integration of in silico and in vitro experiments is a promising research strategy for the discovery of antioxidant peptides from food sources, including edible marine invertebrates. Recently, some research groups have used in silico tools to predict bioactive peptides that can be enzymatically released from selected protein sources and compare relative effectiveness of different proteases. To the authors’ knowledge, the applications of in silico tools in the discovery of potential antioxidant peptides from edible marine invertebrates are scarce. BIOPEP is a database which currently houses 3285 bioactive peptides, including 531 antioxidant peptides (accessed in September 2016) [28]. Darewicz et al. [99] used the BIOPEP database and its computational tools to identify carp proteins that are potential sources of bioactive peptides, in addition to predicting whether those peptides can be released during human gastrointestinal digestion. Their in silico analyses predicted that peptides with 11 types of bioactivity, including antioxidant activity, could be released by carp protein proteolysis. Meanwhile, 13 antioxidant peptides could potentially be released from myosin heavy chain after gastro-duodenal digestion [99], suggesting that the protein is an excellent source of antioxidant peptides. Huang et al. [100] used the BIOPEP database tools to predict the number of antioxidant peptides and other biopeptides that could be released from seven tilapia proteins after in silico proteolysis by using 27 proteases. The study identified myosin heavy chain as the best source of antioxidant peptides. Moreover, chymotrypsin C, ficain, and thermolysin were predicted to be the three most effective among 27 proteases for releasing antioxidant peptides from myosin heavy chain, potentially releasing 13, 13, and 17 antioxidant peptides, respectively, from the protein [100]. Meanwhile, Garcia-Mora et al. [101] used the BIOPEP database to identify antioxidant amino acid sequences harbored within the primary structures of 17 peptides they identified from a pinto bean hydrolysate exhibiting antioxidant activity. On the other hand, in silico analyses involving PeptideRanker, BioPep, and PepBank were used to select five candidates from bioactive peptides identified from donkey milk for chemical synthesis. Further validations of the synthetic peptides lead to the discovery of two novel antioxidant peptides from donkey milk [102]. Considering the aforementioned examples, in silico tools may be a useful set of resources for the discovery of antioxidant peptides from edible marine invertebrates in future. These tools may expedite the screening or pre-selection of protein precursors in edible marine invertebrates for potential sources of bioactive peptides. In silico tools can also be used for searching potential strategies to release bioactive peptides from marine invertebrate proteins [99].

### 6.4. Multifunctionality

Multifunctionality of antioxidant peptides is a potentially productive area of research. As pointed out above, some edible marine invertebrate-derived peptides which showed multiple bioactivities, i.e., WPP, VKP, VKCFR, and GPLGLLGFLGPLGLS [33,42,51], are promising starting points for the design and development of future peptide therapeutics and/or adjunct drugs. When we used antioxidant peptide sequences in Table 2 as queries in the BIOPEP database (accessed in July 2016), we found that QP identified from the blood clam [33] was also reported by others as an inhibitor of dipeptidyl peptidase IV, a potential therapeutic target for the treatment of type 2 diabetes mellitus [103]. SATPdb is a database of therapeutic peptides curated from 22 public domain peptide databases. Analysis of the 19,192 experimentally validated peptide sequences in the database revealed that 39% of the sequences (7512 peptides) have two to three functions [104]. A list of 26 antioxidant peptides with additional experimentally validated bioactivities (e.g., antibacterial, antifungal, anticancer and antihypertensive activities) can be accessed at the SATPdb database (accessed in July 2016) [104]. Thus, it should not be surprising that the antioxidant peptides identified from edible marine invertebrates (Table 2), even many other marine antioxidant peptides yet to be discovered, have multiple functionalities. For example, when we used antioxidant peptide sequences in Table 2 as queries in C2Pred webserver (accessed in July 2016) [105], ten peptide sequences, namely, LPHPSF, YPPAK, WPP, QP, GDQQK, PEASY, VKP, VKCFR, IKK, and FKK, were predicted to be cell-penetrating peptides. Except for GDQQK, the other nine of these peptides were predicted to have anticancer potential by the AntiCP webserver [106]. Despite in silico predictions, experimental validations of the anticancer activity of these peptides and their ability to cross the cellular membrane is necessary in future. Antioxidant peptides that have additional functions, e.g., anticancer and cell-penetrating activity, likely possess greater versatility and commercial value than other antioxidant peptides when it comes to applications in therapy and cosmetics.

### 6.5. Safety

Safety or toxicity assessment of food-derived antioxidant peptides, including those identified from edible marine invertebrates, requires more attention in future. Even when such peptides are identified from food with a long history of human consumption without adverse effects, absence of toxicity and allergenicity in vivo should still be asserted [107,108] before they are used in food, therapy and cosmetics. For the initial screening of potential allergens in food proteins, the European Food Safety Authority (EFSA) recommended the use of in silico tools [109]. A total 2872 peptides identified from hydrolysates of bovine blood globulins were recently assessed in silico for toxicity and allergenicity [110]. Potential toxicity was predicted using the ToxinPred web server [111]. Potential allergenicity of the peptides was assessed by using AlgPred [112] and AllerTOP [113]. In silico analyses predicted that all the peptides were non-toxic, although 564 peptides were predicted to be potential allergens. Such a large-scale screening of peptides for toxicity and allergenicity in the laboratory is predictably expensive. In silico tools represent a less costly and faster strategy to conduct screenings. Such an approach may also be used to narrow down the choices of bioactive peptides to be used for chemical synthesis and further validation of bioactivity, depending on the research objectives. Such a strategy in initial screening for potential toxicity and allergenicity can be adopted in the future search for safe antioxidant peptides from edible marine invertebrates.

When we used the 32 antioxidant peptide sequences listed in Table 2 as input queries in the ToxinPred server, all were predicted to be non-toxic, except for I/L N I/L CCN (accessed in July 2016). The four sequence variations (i.e., INICCN, LNICCN, INLCCN, and LNLCCN) of shortclub cuttlefish-derived antioxidant peptide I/L N I/L CCN [56] were predicted to be toxic. Interestingly, Sudhakar and Nazeer [56] reported that I/L N I/L CCN was not cytotoxic to HT29 cells, which showed greater than 50% viability when exposed to up to 140 µg/mL peptide. Whether the peptide varies in toxicity in different cell types or biological models is an interesting question to address in future. Meanwhile, prediction by ToxinPred or other related in silico tools should also be considered with caution. Such in silico prediction tools, especially when developed primarily based on datasets of bacterial origin, may not always generate predictions that are relevant to the human body [114]. On the other hand, four antioxidant peptides, i.e., LKQELEDLLEKQE [40], NGPLQAGQPGER [41], NADFGLNGLEGLA [43], and GPLGLLGFLGPLGLS [51], were predicted to be allergenic by AlgPred and AllerTOP servers (accessed in July 2016). Such predictions, although still requiring experimental validations, apparently contradict the general assumption that food-derived peptides are safe and non-toxic. Methodologies for the assessment of food peptide toxicity are beyond the scope of this review. For a review on the in vitro, in vivo and in silico tools used for evaluating toxicology of food, the reader is referred to Gosslau [115]. For a comprehensive discussion on empirical and in silico approaches to designing peptides with low toxicity and to predicting peptide toxicity, we refer the reader to Gupta et al. [114].

### 6.6. Need for More Intensive Research on Processing Wastes

Antioxidative protein hydrolysates prepared from by-products/processing wastes of edible marine invertebrates (e.g., squid skin [41,51,116], shrimp waste [54,75,117], shrimp processing wastewater [118], sea cucumber viscera and green sea urchin processing waste [119], scallop gonads [49], and cuttlefish processing wastewater [120]) have been reported. Nevertheless, investigations on such bioresources which culminated in the identification of antioxidant peptide sequences are limited (e.g., see [41,49,51,54]). The antioxidant properties of protein hydrolysates prepared from edible marine invertebrate and their processing by-products were previously reviewed [121,122]. In general, more progresses have been made in the discovery of antioxidant peptides from fish processing by-products than from by-products of edible marine invertebrates. Worldwide catching and processing of shellfish (i.e., cephalopods, bivalves, echinoderms and crustaceans) generate enormous amount of by-products and processing wastes annually [123,124,125,126]. These are protein-rich raw materials which should be tapped into more intensively for the discovery of antioxidant peptides. Meanwhile, more antioxidant peptides have been identified from mussels and oysters, whereas other edible marine invertebrates, such as the lobster, crab, octopus, jellyfish, scallop, abalone, sea cucumber, and sea squirt are underexplored. Future research to valorize shellfish processing by-products should also pay attention to these underexplored species as promising sources of marine antioxidant peptides.

## 7. Conclusions

The purpose of this review was to summarize current progress in the discovery of antioxidant peptides from edible marine invertebrates and to discuss potential applications of the peptides. It is clear from the research reviewed that enzyme-assisted release of peptides from edible marine invertebrates is an effective strategy for the purification and identification of marine antioxidant peptides. A number of edible marine invertebrate-derived peptides exhibiting antioxidant effects in vitro were identified; some of which also demonstrated antioxidant effects in animal models and/or additional bioactivities. Notwithstanding, knowledge gaps exist with regards to the multifunctionality, in vivo stability and safety of edible marine invertebrate-derived peptides. Future research in this direction, supported by the application of bioinformatics tools, should contribute towards realizing potential future applications of these antioxidant peptides in the food, pharmaceutical and cosmetics industries.

## Figures and Tables

**Table 1:** Primary structures of selected antioxidant peptides identified from edible marine invertebrates.

Antioxidant Peptides	References
VKP, VKCFR	[42]
IKK, FKK, FIKK	[48]
HMSY, PEASY	[49]
LWHTH	[50]
LPHPSF	[45]
PIIVYWK, FSVVPSPK, TTANIEDRR	[32]
GPLGLLGFLGPLGLS	[51]

**Table 2:** Selected antioxidant peptides identified from edible marine invertebrates as reported in the literature between years 2000 and 2016.

Species	Protease Used for Hydrolysis *	Antioxidant Parameters Used to Guide Purification and Characterize Purified Peptides	Purification Techniques	Peptide Sequence Identified	Validated with Synthetic Peptides	Reference
Oyster (*Crassostrea madrasensis*)	Papain	DPPH scavenging OH^•^ scavenging # FRAP # Iron chelating # LPI #	UF SPE RP-HPLC	ISIGGQPAGRIVM	×	[36]
Oyster (*Crassostrea gigas*)	In vitro gastrointestinal digestion (Pepsin, Trypsin and α-Chymotrypsin	OH^•^ scavenging O_2_^•−^ scavenging Cellular radical scavenging Protection against OH^•^-induced DNA damage LPI	AEC RP-HPLC(×2)	LKQELEDLLEKQE	×	[40]
Oyster (*Crassostrea talienwhanensis*)	Subtilisin (Alcalase)	DPPH scavenging OH^•^ scavenging	UF SEC RP-HPLC(×2)	PVMGA QHGV	×	[35]
Mussel (*Mytilus coruscus*)	Papain	DPPH scavenging OH^•^ scavenging O_2_^•−^ scavenging Alkyl radical scavenging In vivo antioxidant defense	UF AEC RP-HPLC(×2) GPC	SLPIGLMIAM	×	[44]
Mussel (*Mytilus coruscus*)	In vitro gastrointestinal digestion (Pepsin, Trypsin and α-Chymotrypsin)	LPI OH^•^ scavenging O_2_^•−^ scavenging Carbon-centered radical scavenging	AEC SEC RP-HPLC	LVGDEQAVPAVCVP	×	[52]
Blue mussel (*Mytilus edulis*)	Pepsin	DPPH scavenging ORAC Protection against H_2_O_2_-induced cytotoxicity	UF CEC RP-HPLC(×2)	PIIVYWK TTANIEDRR FSVVPSPK	√	[32]
Blue mussel (*Mytilus edulis*)	Neutrase	DPPH scavenging OH^•^ scavenging O_2_^•−^ scavenging LPI	UF SEC RP-HPLC	YPPAK	×	[31]
Blood Clam (*Tegillarca granosa*)	Neutrase	DPPH scavenging ABTS scavenging OH^•^ scavenging O_2_^•−^ scavenging LPI	UF AEC SEC RP-HPLC	WPP QP	×	[33]
Short-necked Clam (*Ruditapes philippinarum*)	α-Chymotrypsin	DPPH scavenging OH^•^ scavenging Alkyl radicalscavenging O_2_^•−^ scavenging	UF AEC RP-HPLC(×3)	SVEIQALCDM	×	[53]
Short-necked Clam (*Ruditapes philippinarum*)	Trypsin	DPPH scavenging Reducing power Protection against OH^•^-induced DNA damage	UF SEC RP-HPLC	GDQQK	×	[34]
Scallop (*Patinopecten yessoensis*)	Neutrase	DPPH scavenging # OH^•^ scavenging Iron chelating # Reducing power # Protection against OH^•^-induced DNA damage	SEC	HMSY PEASY	√	[49]
Jellyfish (*Rhopilema esculentum*)	Alcalase	OH^•^ scavenging Protection against H_2_O_2_-induced cytotoxicity Cellular antioxidant enzyme activity	UF AEC RP-HPLC	VKP VKCFR	√	[42]
Jumbo squid (*Dosidicus gigas*)	Trypsin	LPI OH^•^ scavenging Carbon-centered radical scavenging Iron chelating # Protection against *t*-butyl hydroperoxide-induced cytotoxicity	UF CEC SEC RP-HPLC	FDSGPAGVL NGPLQAGQPGER	×	[41]
Giant squid (*Dosidicus gigas*)	Trypsin	LPI OH^•^ scavenging O_2_^•−^ scavenging Carbon-centered radical scavenging Protection against *t*-butyl hydroperoxide-induced cytotoxicity	UF CEC SEC RP-HPLC(×2)	NADFGLNGLEGLA NGLEGLK	×	[43]
Giant squid (*Dosidicus gigas*) (skin)	Alcalase	ABTS scavenging FRAP	UF SEC	GPLGLLGFLGPLGLS	√	[51]
Shortclub cuttlefish (*Sepia brevimana*)	Trypsin	DPPH scavenging ABTS scavenging # O_2_^•−^ scavenging # Total antioxidant capacity # Reducing power Iron chelating # LPI Protection against OH^•^-induced DNA damage	AEC SEC	I/L N I/L CCN	×	[56]
Indian squid (*Loligo duvauceli*)	α-chymotrypsin	DPPH scavenging OH^•^ scavenging O_2_^•−^ scavenging # Reducing power Iron chelating LPI Protection against OH^•^-induced DNA damage Cellular radical scavenging	AEC SEC	WCTSVS	×	[57]
Prawn (*Penaeus japonicus*)	Pepsin	LPI	SEC CEC RP-HPLC	IKK FKK FIKK	√	[48]
Shrimp processing by-products	Alcalase	DPPH scavenging	Methanol extraction CEC SEC RP-HPLC(×2)	SVAMLFH	×	[54]
Sea cucumber (*Stichopus japonicus*)	Trypsin	OH^•^ scavenging O_2_^•−^ scavenging	SEC AEC SEC RP-HPLC	GPEPTGPT GAPQWLR	×	[55]
Sea squirt (*Styela clava*)	Pepsin	Peroxyl radical scavenging	SEC RP-HPLC	LWHTH	√	[50]
Sea squirt (*Styela plicata*)	Trypsin	Peroxyl radical scavenging DPPH scavenging OH^•^ scavenging Cellular radical scavenging Protection against APPH-induced cytotoxicity Protection against APPH-induced ROS generation and cell death in zebrafish embryos	AEC SEC RP-HPLC	LPHPSF	√	[45]

* Only the protease which was associated with the peptide sequence identified is listed. # Not tested with purified or synthetic peptides. AEC, anion exchange chromatography; CEC, cation exchange chromatography; FRAP, Ferric Reducing Antioxidant Power; LPI, lipid peroxidation inhibition; RP-HPLC(×2), two-step RP-HPLC; RP-HPLC(×3), three-step RP-HPLC; SEC, size exclusion chromatography; SPE, solid phase extraction; UF, ultrafiltration membrane; ×, not validated; √, validated.

**Table 3:** Molecular masses of 32 antioxidant peptides identified from edible marine invertebrates.

Antioxidant Peptides	Molecular Mass (Da)	References
QP	243.23	[33]
VKP	342	[42]
IKK	388	[48]
WPP	398.44	[33]
FKK	422	[48]
QHGV	440	[35]
PVMGA	518	[35]
FIKK	535	[48]
HMSY	536.16	[49]
PEASY	565.21	[49]
YPPAK	574	[31]
GDQQK	574.27 *	[34]
VKCFR	651	[42]
I/L N I/L CCN	679.5	[56]
WCTSVS	682.5	[57]
LWHTH	692.2	[50]
LPHPSF	696.3	[45]
NGLEGLK	747	[43]
SVAMLFH	804.4	[54]
FSVVPSPK	860.09	[32]
FDSGPAGVL	880.18	[41]
PIIVYWK	1004.57	[32]
SLPIGLMIAM	1044.57 *	[44]
TTANIEDRR	1074.54	[32]
SVEIQALCDM	1107.49 *	[53]
NGPLQAGQPGER	1241.59	[41]
ISIGGQPAGRIVM	1297.72	[36]
NADFGLNGLEGLA	1307	[43]
GPLGLLGFLGPLGLS	1409.63 **	[51]
GPEPTGPTGAPQWLR	1563	[55]
LVGDEQAVPAVCVP	1590	[52]
LKQELEDLLEKQE	1600	[40]

* Calculated online using PepDraw [63]; ** Calculated from the *m*/*z* value reported.

**Table 4:** Percentages of hydrophobic residues in 15 edible marine invertebrate-derived antioxidant peptides, which exhibited lipid peroxidation inhibitory activity.

Antioxidant Peptides	Hydrophobic Amino Acid Residue (%) *	References
NGLEGLK	28.57	[43]
LKQELEDLLEKQE	30.77	[40]
NGPLQAGQPGER	33.33	[41]
IKK	33.33	[48]
FKK	33.33	[48]
WCTSVS	33.33	[57]
I/L N I/L CCN	33.33	[56]
FDSGPAGVL	44.44	[41]
NADFGLNGLEGLA	46.15	[43]
QP	50	[33]
FIKK	50	[48]
ISIGGQPAGRIVM	53.85	[36]
YPPAK	60	[31]
LVGDEQAVPAVCVP	64.29	[52]
WPP	100	[33]

* Percentages of hydrophobic residues were computed manually, based on the classification of A, I, L, M, F, P, W, and V as hydrophobic amino acids (The IARCTP53 Database [65]).

**Table 5:** Percentages of hydrophobic residues in 13 edible marine invertebrate-derived antioxidant peptides, whose activities were confirmed using pure synthetic peptides.

Antioxidant Peptides	Hydrophobic Amino Acid Residue (%) *	References
TTANIEDRR	22.22	[32]
HMSY	25	[49]
IKK	33.33	[48]
FKK	33.33	[48]
PEASY	40	[49]
LWHTH	40	[50]
VKCFR	40	[42]
FIKK	50	[48]
GPLGLLGFLGPLGLS	60	[51]
FSVVPSPK	62.5	[32]
VKP	66.67	[42]
LPHPSF	66.67	[45]
PIIVYWK	71.43	[32]

* Percentages of hydrophobic residues were computed manually, based on the classification of A, I, L, M, F, P, W, and V as hydrophobic amino acids (The IARCTP53 Database [65]).

## Supplementary Materials

The following are available online at www.mdpi.com/1660-3397/15/2/42/s1. Figure S1: The trends in the numbers of publications in the field of antioxidant peptides over the past 24 years, based on the Scopus database (accessed in November 2016). Input query used was “antioxidant peptide” OR “antioxidative peptide”.

## References

[1] Weidinger A., Kozlov A. (2015). Biological activities of reactive oxygen and nitrogen species: Oxidative stress versus signal transduction. Biomolecules.

[2] Ye Z.-W., Zhang J., Townsend D.M., Tew K.D. (2015). Oxidative stress, redox regulation and diseases of cellular differentiation. BBA-Gen. Subj..

[3] Luca M., Luca A., Calandra C. (2015). The role of oxidative damage in the pathogenesis and progression of Alzheimer’s disease and vascular dementia. Oxid. Med. Cell. Longev..

[4] Chakrabarti S., Jahandideh F., Wu J. (2014). Food-Derived bioactive peptides on inflammation and oxidative stress. Biomed. Res. Int..

[5] Reeg S., Grune T. (2015). Protein oxidation in aging: Does it play a role in aging progression?. Antioxid. Redox Signal..

[6] Miyake Y., Fukushima W., Tanaka K., Sasaki S., Kiyohara C., Tsuboi Y., Yamada T., Oeda T., Miki T., Kawamura N. (2011). Dietary intake of antioxidant vitamins and risk of Parkinson’s disease: A case–control study in Japan. Eur. J. Neurol..

[7] Bo Y., Lu Y., Zhao Y., Zhao E., Yuan L., Lu W., Cui L., Lu Q. (2016). Association between dietary vitamin C intake and risk of esophageal cancer: A dose-response meta-analysis. Int. J. Cancer.

[8] Aboonabi A., Singh I. (2016). The effectiveness of antioxidant therapy in aspirin resistance, diabetes population for prevention of thrombosis. Biomed. Pharmacother..

[9] Tamay-Cach F., Quintana-Pérez J.C., Trujillo-Ferrara J.G., Cuevas-Hernández R.I., Del Valle-Mondragón L., García-Trejo E.M., Arellano-Mendoza M.G. (2016). A review of the impact of oxidative stress and some antioxidant therapies on renal damage. Ren. Fail..

[10] Bielli A., Scioli M.G., Mazzaglia D., Doldo E., Orlandi A. (2015). Antioxidants and vascular health. Life Sci..

[11] Zhong Y., Shahidi F., Shahidi F. (2015). Methods for the assessment of antioxidant activity in foods. Handbook of Antioxidants for Food Preservation.

[12] Agyei D., Danquah M.K., Sarethy I.P., Pan S., Rani V., Yadav S.U.C. (2015). Antioxidative peptides derived from food proteins. Free Radicals in Human Health and Disease.

[13] Sarmadi B.H., Ismail A. (2010). Antioxidative peptides from food proteins: A review. Peptides.

[14] Sampath Kumar N.S., Nazeer R.A., Jaiganesh R. (2011). Purification and identification of antioxidant peptides from the skin protein hydrolysate of two marine fishes, horse mackerel (*Magalaspis cordyla*) and croaker (*Otolithes ruber*). Amino Acids.

[15] Cheung R.C.F., Ng T.B., Wong J.H. (2015). Marine peptides: Bioactivities and applications. Mar. Drugs.

[16] Ghosh S. (2016). Peptide therapeutics market: Forecast and analysis 2015–2025. Chim. Oggi Chem. Today.

[17] Fosgerau K., Hoffmann T. (2015). Peptide therapeutics: Current status and future directions. Drug Discov. Today.

[18] Sila A., Bougatef A. (2016). Antioxidant peptides from marine by-products: Isolation, identification and application in food systems. A review. J. Funct. Foods.

[19] Lemes A.C., Sala L., Ores J.d.C., Braga A.R.C., Egea M.B., Fernandes K.F. (2016). A review of the latest advances in encrypted bioactive peptides from protein-rich waste. Int. J. Mol. Sci..

[20] Ngo D.-H., Vo T.-S., Ngo D.-N., Wijesekara I., Kim S.-K. (2012). Biological activities and potential health benefits of bioactive peptides derived from marine organisms. Int. J. Biol. Macromol..

[21] Samaranayaka A.G.P., Li-Chan E.C.Y. (2011). Food-derived peptidic antioxidants: A review of their production, assessment, and potential applications. J. Funct. Foods.

[22] Xiong Y.L., Mine Y., Li-Chan E., Jiang B. (2010). Antioxidant peptides. Bioactive Proteins and Peptides as Functional Foods and Nutraceuticals.

[23] Kim S.-K., Wijesekara I., Kim S.-K. (2013). Marine-Derived Peptides: Development and Health Prospects. Marine Proteins and Peptides: Biological Activities and Applications.

[24] Kim S.-K., Wijesekara I. (2010). Development and biological activities of marine-derived bioactive peptides: A review. J. Funct. Foods.

[25] Li Y., Yu J. (2015). Research progress in structure-activity relationship of bioactive peptides. J. Med. Food.

[26] Zou T.-B., He T.-P., Li H.-B., Tang H.-W., Xia E.-Q. (2016). The structure-activity relationship of the antioxidant peptides from natural proteins. Molecules.

[27] Scopus. http://www.scopus.com.

[28] Minkiewicz P., Dziuba J., Iwaniak A., Dziuba M., Darewicz M. (2008). BIOPEP database and other programs for processing bioactive peptide sequences. J. AOAC Int..

[29] Halim N.R.A., Yusof H.M., Sarbon N.M. (2016). Functional and bioactive properties of fish protein hydolysates and peptides: A comprehensive review. Trends Food Sci. Technol..

[30] Senevirathne M., Kim S.-K. (2012). Development of bioactive peptides from fish proteins and their health promoting ability. Adv. Food Nutr. Res..

[31] Wang B., Li L., Chi C.F., Ma J.H., Luo H.Y., Xu Y.F. (2013). Purification and characterisation of a novel antioxidant peptide derived from blue mussel (*Mytilus edulis*) protein hydrolysate. Food Chem..

[32] Park S.Y., Kim Y.S., Ahn C.B., Je J.Y. (2016). Partial purification and identification of three antioxidant peptides with hepatoprotective effects from blue mussel (*Mytilus edulis*) hydrolysate by peptic hydrolysis. J. Funct. Foods.

[33] Chi C.F., Hu F.Y., Wang B., Li T., Ding G.F. (2015). Antioxidant and anticancer peptides from the protein hydrolysate of blood clam (*Tegillarca granosa*) muscle. J. Funct. Foods.

[34] Li R., Yang Z.S., Sun Y., Li L., Wang J.B., Ding G. (2015). Purification and antioxidant property of antioxidative oligopeptide from short-necked clam (*Ruditapes philippinarum*) hydrolysate in vitro. J. Aquat. Food Prod. Technol..

[35] Wang Q., Li W., He Y., Ren D., Kow F., Song L., Yu X. (2014). Novel antioxidative peptides from the protein hydrolysate of oysters (*Crassostrea talienwhanensis*). Food Chem..

[36] Asha K.K., Remya Kumari K.R., Ashok Kumar K., Chatterjee N.S., Anandan R., Mathew S. (2016). Sequence determination of an antioxidant peptide obtained by enzymatic hydrolysis of oyster *Crassostrea madrasensis* (Preston). Int. J. Pept. Res. Ther..

[37] Jung W.K., Rajapakse N., Kim S.K. (2005). Antioxidative activity of a low molecular weight peptide derived from the sauce of fermented blue mussel, Mytilus edulis. Eur. Food Res. Technol..

[38] Rajapakse N., Mendis E., Jung W.K., Je J.Y., Kim S.K. (2005). Purification of a radical scavenging peptide from fermented mussel sauce and its antioxidant properties. Food Res. Int..

[39] Kleekayai T., Harnedy P.A., O’Keeffe M.B., Poyarkov A.A., CunhaNeves A., Suntornsuk W., FitzGerald R.J. (2015). Extraction of antioxidant and ACE inhibitory peptides from Thai traditional fermented shrimp pastes. Food Chem..

[40] Qian Z.-J., Jung W.-K., Byun H.-G., Kim S.-K. (2008). Protective effect of an antioxidative peptide purified from gastrointestinal digests of oyster, *Crassostrea gigas* against free radical induced DNA damage. Bioresour. Technol..

[41] Mendis E., Rajapakse N., Byun H.-G., Kim S.-K. (2005). Investigation of jumbo squid (*Dosidicus gigas*) skin gelatin peptides for their in vitro antioxidant effects. Life Sci..

[42] Li J., Li Q., Li J., Zhou B. (2014). Peptides derived from *Rhopilema esculentum* hydrolysate exhibit angiotensin converting enzyme (ACE) inhibitory and antioxidant abilities. Molecules.

[43] Rajapakse N., Mendis E., Byun H.-G., Kim S.-K. (2005). Purification and in vitro antioxidative effects of giant squid muscle peptides on free radical-mediated oxidative systems. J. Nutr. Biochem..

[44] Kim E.-K., Oh H.-J., Kim Y.-S., Hwang J.-W., Ahn C.-B., Lee J.S., Jeon Y.-J., Moon S.-H., Sung S.H., Jeon B.-T. (2013). Purification of a novel peptide derived from *Mytilus coruscus* and in vitro/in vivo evaluation of its bioactive properties. Fish Shellfish Immunol..

[45] Ko S.C., Kim E.A., Jung W.K., Kim W.S., Lee S.C., Son K.T., Kim J.I., Jeon Y.J. (2016). A hexameric peptide purified from *Styela plicata* protects against free radical-induced oxidative stress in cells and zebrafish model. RSC Adv..

[46] Wu R.B., Wu C.L., Liu D., Yang X.H., Huang J.F., Zhang J., Liao B., He H.L., Li H. (2015). Overview of antioxidant peptides derived from marine resources: The sources, characteristic, purification, and evaluation methods. Appl. Biochem. Biotechnol..

[47] Ngo D.-H., Kim S.-K. (2013). Marine bioactive peptides as potential antioxidants. Curr. Protein Pept. Sci..

[48] Suetsuna K. (2000). Antioxidant peptides from the protease digest of prawn (*Penaeus japonicus*) muscle. Mar. Biotechnol..

[49] Wu H.T., Jin W.G., Sun S.G., Li X.S., Duan X.H., Li Y., Yang Y.T., Han J.R., Zhu B.W. (2016). Identification of antioxidant peptides from protein hydrolysates of scallop (*Patinopecten yessoensis*) female gonads. Eur. Food Res. Technol..

[50] Kang N., Ko S.C., Samarakoon K., Kim E.A., Kang M.C., Lee S.C., Kim J., Kim Y.T., Kim J.S., Kim H. (2013). Purification of antioxidative peptide from peptic hydrolysates of Mideodeok (*Styela clava*) flesh tissue. Food Sci. Biotechnol..

[51] Alemán A., Giménez B., Pérez-Santin E., Gómez-Guillén M.C., Montero P. (2011). Contribution of Leu and Hyp residues to antioxidant and ACE-inhibitory activities of peptide sequences isolated from squid gelatin hydrolysate. Food Chem..

[52] Jung W.-K., Qian Z.-J., Lee S.-H., Choi S.Y., Sung N.J., Byun H.-G., Kim S.-K. (2007). Free radical scavenging activity of a novel antioxidative peptide isolated from in vitro gastrointestinal digests of *Mytilus coruscus*. J. Med. Food.

[53] Kim E.K., Hwang J.W., Kim Y.S., Ahn C.B., Jeon Y.J., Kweon H.J., Bahk Y.Y., Moon S.H., Jeon B.T., Park P.J. (2013). A novel bioactive peptide derived from enzymatic hydrolysis of *Ruditapes philippinarum*: Purification and investigation of its free-radical quenching potential. Process Biochem..

[54] Zhao J., Huang G., Jiang J. (2013). Purification and characterization of a new DPPH radical scavenging peptide from shrimp processing by-products hydrolysate. J. Aquat. Food Prod. Technol..

[55] Zhou X., Wang C., Jiang A. (2012). Antioxidant peptides isolated from sea cucumber *Stichopus Japonicus*. Eur. Food Res. Technol..

[56] Sudhakar S., Nazeer R.A. (2015). Preparation of potent antioxidant peptide from edible part of shortclub cuttlefish against radical mediated lipid and DNA damage. LWT-Food Sci. Technol..

[57] Sudhakar S., Nazeer R.A. (2015). Structural characterization of an Indian squid antioxidant peptide and its protective effect against cellular reactive oxygen species. J. Funct. Foods.

[58] Grienke U., Silke J., Tasdemir D. (2014). Bioactive compounds from marine mussels and their effects on human health. Food Chem..

[59] Pérez-Vega J.A., Olivera-Castillo L., Gómez-Ruiz J.T., Hernández-Ledesma B. (2013). Release of multifunctional peptides by gastrointestinal digestion of sea cucumber (*Isostichopus badionotus*). J. Funct. Foods.

[60] Amarowicz R., Shahidi F. (1997). Antioxidant activity of peptide fractions of capelin protein hydrolysates. Food Chem..

[61] Ngo D.-H., Wijesekara I., Vo T.-S., Van Ta Q., Kim S.-K. (2011). Marine food-derived functional ingredients as potential antioxidants in the food industry: An overview. Food Res. Int..

[62] Zhuang H., Tang N., Yuan Y. (2013). Purification and identification of antioxidant peptides from corn gluten meal. J. Funct. Foods.

[63] PepDraw. http://www.tulane.edu/~biochem/WW/PepDraw/.

[64] Harnedy P.A., FitzGerald R.J. (2012). Bioactive peptides from marine processing waste and shellfish: A review. J. Funct. Foods.

[65] IARC TP53 Database. http://p53.iarc.fr/AAProperties.aspx.

[66] Chan K.M., Decker E.A., Feustman C. (1994). Endogenous skeletal muscle antioxidants. Crit. Rev. Food Sci. Nutr..

[67] Dhaval A., Yadav N., Purwar S. (2016). Potential applications of food derived bioactive peptides in management of health. Int. J. Pept. Res. Ther..

[68] Wang B., Wang Y.-M., Chi C.-F., Luo H.-Y., Deng S.-G., Ma J.-Y. (2013). Isolation and characterization of collagen and antioxidant collagen peptides from scales of croceine croaker (*Pseudosciaena crocea*). Mar. Drugs.

[69] Thorkelsson G., Kristinsson H.G. (2009). Bioactive Peptides from Marine Sources. State of Art. Report to the NORA Fund.

[70] Hartmann R., Meisel H. (2007). Food-Derived peptides with biological activity: From research to food applications. Curr. Opin. Biotechnol..

[71] Dziuba B., Dziuba M. (2014). Milk proteins-derived bioactive peptides in dairy products: Molecular, biological and methodological aspects. Acta Sci. Pol. Technol. Aliment..

[72] Freitas A.C., Andrade J.C., Silva F.M., Rocha-Santos T.A.P., Duarte A.C., Gomes A.M. (2013). Antioxidative peptides: Trends and perspectives for future research. Curr. Med. Chem..

[73] Sivaraman B., Shakila R.J., Jeyasekaran G., Sukumar D., Manimaran U., Sumathi G. (2016). Antioxidant activities of squid protein hydrolysates prepared with papain using response surface methodology. Food Sci. Biotechnol..

[74] Jridi M., Lassoued I., Nasri R., Ayadi M.A., Nasri M., Souissi N. (2014). Characterization and potential use of cuttlefish skin gelatin hydrolysates prepared by different microbial proteases. Biomed. Res. Int..

[75] Dey S.S., Dora K.C. (2014). Antioxidative activity of protein hydrolysate produced by alcalase hydrolysis from shrimp waste (*Penaeus monodon* and *Penaeus indicus*). J. Food Sci. Technol. (Mysore).

[76] Nikoo M., Benjakul S., Ehsani A., Li J., Wu F., Yang N., Xu B., Jin Z., Xu X. (2014). Antioxidant and cryoprotective effects of a tetrapeptide isolated from Amur sturgeon skin gelatin. J. Funct. Foods.

[77] Nikoo M., Regenstein J.M., Ghomi M.R., Benjakul S., Yang N., Xu X. (2015). Study of the combined effects of a gelatin-derived cryoprotective peptide and a non-peptide antioxidant in a fish mince model system. LWT-Food Sci. Technol..

[78] Aluko R.E., Shahidi F. (2015). Amino acids, peptides, and proteins as antioxidants for food preservation. Handbook of Antioxidants for Food Preservation.

[79] Cho J., Won K., Wu D., Soong Y., Liu S., Szeto H.H., Hong M.K. (2007). Potent mitochondria-targeted peptides reduce myocardial infarction in rats. Coron. Artery Dis..

[80] Hou Y., Li S., Wu M., Wei J., Ren Y., Du C., Wu H., Han C., Duan H., Shi Y. (2016). Mitochondria-Targeted peptide SS-31 attenuates renal injury via an antioxidant effect in diabetic nephropathy. Am. J. Physiol. Ren. Physiol..

[81] Huang J., Li X., Li M., Li J., Xiao W., Ma W., Chen X., Liang X., Tang S., Luo Y. (2013). Mitochondria-targeted antioxidant peptide SS31 protects the retinas of diabetic rats. Curr. Mol. Med..

[82] Righi V., Constantinou C., Mintzopoulos D., Khan N., Mupparaju S.P., Rahme L.G., Swartz H.M., Szeto H.H., Tompkins R.G., Tzika A.A. (2013). Mitochondria-targeted antioxidant promotes recovery of skeletal muscle mitochondrial function after burn trauma assessed by in vivo 31P nuclear magnetic resonance and electron paramagnetic resonance spectroscopy. FASEB J..

[83] Lee H.Y., Kaneki M., Andreas J., Tompkins R.G., Martyn J.A.J. (2011). Novel mitochondria-targeted antioxidant peptide ameliorates burn-induced apoptosis and endoplasmic reticulum stress in the skeletal muscle of mice. Shock.

[84] Homayouni-Tabrizi M., Asoodeh A., Abbaszadegan M.R., Shahrokhabadi K., Nakhaie Moghaddam M. (2015). An identified antioxidant peptide obtained from ostrich (*Struthio camelus*) egg white protein hydrolysate shows wound healing properties. Pharm. Biol..

[85] Lintner K., Draelos Z.D. (2015). Peptides and proteins. Cosmetic Dermatology: Products and Procedures.

[86] Reddy B., Jow T., Hantash B.M. (2012). Bioactive oligopeptides in dermatology: Part I. Exp. Dermatol..

[87] Reddy B.Y., Jow T., Hantash B.M. (2012). Bioactive oligopeptides in dermatology: Part II. Exp. Dermatol..

[88] Zhang L., Falla T.J. (2009). Cosmeceuticals and peptides. Clin. Dermatol..

[89] Pickart L., Vasquez-Soltero J., Margolina A. (2015). GHK-Cu may prevent oxidative stress in skin by regulating copper and modifying expression of numerous antioxidant genes. Cosmetics.

[90] Sonthalia S., Daulatabad D., Sarkar R. (2016). Glutathione as a skin whitening agent: Facts, myths, evidence and controversies. Indian J. Dermatol. Venereol. Leprol..

[91] Shen S., Chahal B., Majumder K., You S.-J., Wu J. (2010). Identification of novel antioxidative peptides derived from a thermolytic hydrolysate of ovotransferrin by LC-MS/MS. J. Agric. Food. Chem..

[92] Jahandideh F., Chakrabarti S., Davidge S.T., Wu J. (2016). Antioxidant peptides identified from ovotransferrin by the ORAC method did not show anti-inflammatory and antioxidant activities in endothelial cells. J. Agric. Food. Chem..

[93] Tenore G.C., Ritieni A., Campiglia P., Stiuso P., Di Maro S., Sommella E., Pepe G., D’Urso E., Novellino E. (2015). Antioxidant peptides from “Mozzarella di Bufala Campana DOP” after simulated gastrointestinal digestion: In vitro intestinal protection, bioavailability, and anti-haemolytic capacity. J. Funct. Foods.

[94] Fernández-Musoles R., Salom J.B., Castelló-Ruiz M., Contreras M.d.M., Recio I., Manzanares P. (2013). Bioavailability of antihypertensive lactoferricin B-derived peptides: Transepithelial transport and resistance to intestinal and plasma peptidases. Int. Dairy J..

[95] Renukuntla J., Vadlapudi A.D., Patel A., Boddu S.H.S., Mitra A.K. (2013). Approaches for enhancing oral bioavailability of peptides and proteins. Int. J. Pharm..

[96] Bruno B.J., Miller G.D., Lim C.S. (2013). Basics and recent advances in peptide and protein drug delivery. Ther. Deliv..

[97] Vermeirssen V., Camp J.V., Verstraete W. (2004). Bioavailability of angiotensin I converting enzyme inhibitory peptides. Br. J. Nutr..

[98] Bhattacharyya A., Chattopadhyay R., Mitra S., Crowe S.E. (2014). Oxidative stress: An essential factor in the pathogenesis of gastrointestinal mucosal diseases. Physiol. Rev..

[99] Darewicz M., Borawska J., Pliszka M. (2016). Carp proteins as a source of bioactive peptides—An in silico approach. Czech J. Food Sci..

[100] Huang B.B., Lin H.C., Chang Y.W. (2015). Analysis of proteins and potential bioactive peptides from tilapia (*Oreochromis* spp.) processing co-products using proteomic techniques coupled with BIOPEP database. J. Funct. Foods.

[101] Garcia-Mora P., Peñas E., Frias J., Zieliński H., Wiczkowski W., Zielińska D., Martínez-Villaluenga C. (2016). High-pressure-assisted enzymatic release of peptides and phenolics increases angiotensin converting enzyme I inhibitory and antioxidant activities of pinto bean hydrolysates. J. Agric. Food. Chem..

[102] Zenezini Chiozzi R., Capriotti A.L., Cavaliere C., La Barbera G., Piovesana S., Samperi R., Laganà A. (2016). Purification and identification of endogenous antioxidant and ACE-inhibitory peptides from donkey milk by multidimensional liquid chromatography and nanoHPLC-high resolution mass spectrometry. Anal. Bioanal. Chem..

[103] Hikida A., Ito K., Motoyama T., Kato R., Kawarasaki Y. (2013). Systematic analysis of a dipeptide library for inhibitor development using human dipeptidyl peptidase IV produced by a *Saccharomyces cerevisiae* expression system. Biochem. Biophys. Res. Commun..

[104] Singh S., Chaudhary K., Dhanda S.K., Bhalla S., Usmani S.S., Gautam A., Tuknait A., Agrawal P., Mathur D., Raghava G.P.S. (2015). SATPdb: A database of structurally annotated therapeutic peptides. Nucleic Acids Res..

[105] Tang H., Su Z.-D., Wei H.-H., Chen W., Lin H. (2016). Prediction of cell-penetrating peptides with feature selection techniques. Biochem. Biophys. Res. Commun..

[106] Tyagi A., Kapoor P., Kumar R., Chaudhary K., Gautam A., Raghava G.P.S. (2013). In silico models for designing and discovering novel anticancer peptides. Sci. Rep..

[107] Hartmann R., Wal J.M., Bernard H., Pentzien A.K. (2007). Cytotoxic and allergenic potential of bioactive proteins and peptides. Curr. Pharm. Des..

[108] Schaafsma G. (2009). Safety of protein hydrolysates, fractions thereof and bioactive peptides in human nutrition. Eur. J. Clin. Nutr..

[109] Christer H., Andersson S., Arpaia D., Casacuberta J., Davies H., Jardin P., Flachowsky G. (2010). Scientific opinion on the assessment of allergenicity of GM plants and microorganisms and derived food and feed. EFSA J..

[110] Lafarga T., Wilm M., Wynne K., Hayes M. (2016). Bioactive hydrolysates from bovine blood globulins: Generation, characterisation, and in silico prediction of toxicity and allergenicity. J. Funct. Foods.

[111] Gupta S., Kapoor P., Chaudhary K., Gautam A., Kumar R., Raghava G.P.S. (2013). In silico approach for predicting toxicity of peptides and proteins. PLoS ONE.

[112] Saha S., Raghava G.P.S. (2006). AlgPred: Prediction of allergenic proteins and mapping of IgE epitopes. Nucleic Acids Res..

[113] Dimitrov I., Bangov I., Flower D.R., Doytchinova I. (2014). AllerTOP v.2—A server for in silico prediction of allergens. J. Mol. Model..

[114] Gupta S., Kapoor P., Chaudhary K., Gautam A., Kumar R., Raghava G.P.S., Zhou P., Huang J. (2015). Peptide toxicity prediction. Computational Peptidology.

[115] Gosslau A. (2016). Assessment of food toxicology. Food Sci. Hum. Wellness.

[116] Nakchum L., Kim S.M. (2016). Preparation of squid skin collagen hydrolysate as an antihyaluronidase, antityrosinase, and antioxidant agent. Prep. Biochem. Biotechnol..

[117] Vieira M.A., Oliveira D.D., Kurozawa L.E. (2016). Production of peptides with radical scavenging activity and recovery of total carotenoids using enzymatic protein hydrolysis of shrimp waste. J. Food Biochem..

[118] Tonon R.V., Dos Santos B.A., Couto C.C., Mellinger-Silva C., Brígida A.I.S., Cabral L.M.C. (2016). Coupling of ultrafiltration and enzymatic hydrolysis aiming at valorizing shrimp wastewater. Food Chem..

[119] Mamelona J., Saint-Louis R., Pelletier É. (2010). Nutritional composition and antioxidant properties of protein hydrolysates prepared from echinoderm byproducts. Int. J. Food Sci. Technol..

[120] Amado I.R., Vázquez J.A., González M.P., Murado M.A. (2013). Production of antihypertensive and antioxidant activities by enzymatic hydrolysis of protein concentrates recovered by ultrafiltration from cuttlefish processing wastewaters. Biochem. Eng. J..

[121] Giménez B., López-Caballero E.M., Montero P.M., Gómez-Guillén C.M. (2012). Antioxidant peptides from marine origin: Sources, properties and potential applications. Antioxidant Polymers: Synthesis, Properties, and Applications.

[122] Lee J.K., Jeon J.K., Kim S.K., Byun H.G. (2012). Characterization of bioactive peptides obtained from marine invertebrates. Adv. Food Nutr. Res..

[123] Yan N., Chen X. (2015). Sustainability: Don’t waste seafood waste. Nature.

[124] Food and Agriculture Organization of the United Nations (FAO) (2014). The State of World Fisheries and Aquaculture 2014. Opportunities and Challenges.

[125] Food and Agriculture Organization of the United Nations (FAO) (2016). The State of World Fisheries and Aquaculture 2016. Contributing to Food Security and Nutrition for All.

[126] Olsen R.L., Toppe J., Karunasagar I. (2014). Challenges and realistic opportunities in the use of by-products from processing of fish and shellfish. Trends Food Sci. Technol..

