# Supplementary material for: Enzyme-Assisted Discovery of Antioxidant Peptides from Edible Marine Invertebrates: A Review
Source: Mar Drugs. 2017 Feb 16;15(2):42. doi: 10.3390/md15020042 (PMC5334622; doi:10.3390/md15020042)
Supplement: Supplementary file 1 [file marinedrugs-15-00042-s001.pdf]

Supplementary Materials: Enzyme-Assisted Discovery of Antioxidant Peptides from Edible Marine Invertebrates: A Review

Tsun-Thai Chai, Yew-Chye Law, Fai-Chu Wong and Se-Kwon Kim

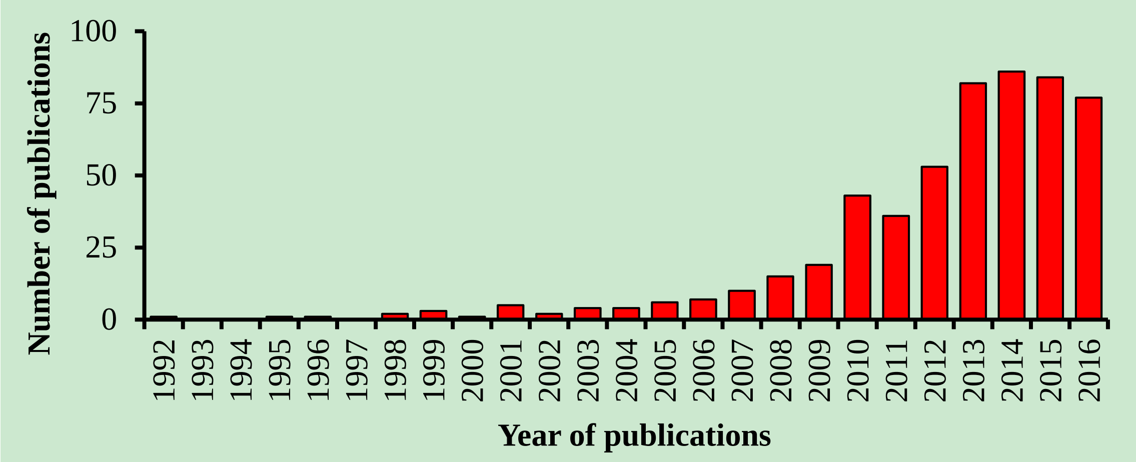

Figure S1. The trends in the numbers of publications in the field of antioxidant peptides over the past 24 years, based on the Scopus database (accessed in November 2016). Input query used was "antioxidant peptide" OR "antioxidative peptide".
